# Supplementary material for: Leveraging PSO-MLP for intelligent assessment of student learning in remote environments: a multimodal approach
Source: PeerJ Comput Sci. 2025 Aug 18;11:e3121. doi: 10.7717/peerj-cs.3121 (PMC12453797; doi:10.7717/peerj-cs.3121)
Supplement: Supplemental Information 1 [file peerj-cs-11-3121-s001.zip › Intelligent Assessment-code/Report/CI_Project_Report.pdf]

# CI Course Project

*EEG Signal Classification*

Mohammad Amin Alamalhoda

Sharif University of Technology $\mathcal{SUT}$

# Contents


1 Git and Project Dependencies . . . 1
1.1 Git . . . 1
1.2 Project Dependencies . . . 1
2 Datas . . . 2
3 Features . . . 3
3.1 Mean Frequency . . . 3
3.2 Median Frequency . . . 3
3.3 Total Power of Channels . . . 3
3.4 Powers of Delta, Theta, Alpha, and Beta Frequency Bands . . . 3
3.5 Entropy . . . 3
3.6 Lyapunov Exponent . . . 3
3.7 Average of Differentiate of Trials . . . 3
3.8 Skewness . . . 3
3.9 Kurtosis . . . 3
3.10 Phase of the coefficients of the FFT . . . 3
4 Fisher Score . . . 4
5 Processing the Datas . . . 4

# List of Figures


1 Amplitude and STFT of Average of all Trials and all Channels . . . . . . . . . . . . . . . . . . . . 2
2 Amplitude and STFT of Average of all Trials and all Channels Before and After CSP . . . . . . 4

# List of Tables

# 1 Git and Project Dependencies

## 1.1 Git

This Project is open source and is published on Github. You can watch it using this link.

You can use the following bash command for cloning this project:

```
$ git clone https://github.com/MohammadAminAlamalhoda/EEG-Classification
```

If you don't have `git` installed on your device, you can use the following bash command:

- Linux

  ```
  $ sudo apt-get install git
  ```

- MacOS

  MacOS already have git installed, check its version using bash command below:

  ```
  $ git --version
  ```

  If you uninstalled it, you can install it using `brew`:

  ```
  $ brew install git
  ```

- Windows

  You can download source code of git and make-install it using this link.

## 1.2 Project Dependencies

This project needs the following stuff in order to be compiled successfully.

- Python 3.7/3.8/3.9 Kernel
- Matlab
- PyTorch - Python Lib

You can install the required python dependencies by running:

```
$ pip install -r requirements.txt
```

## 2 Datas

I loaded the datas and plotted the amplitude and STFT of average of all the channels and trials. You can see them in Figure 2.

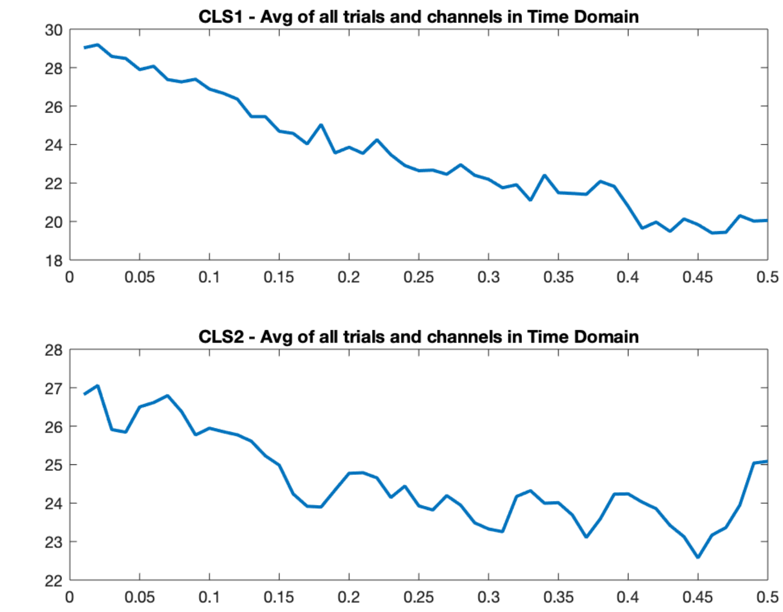


(a) Amplitude of Average of all Trials and all Channels

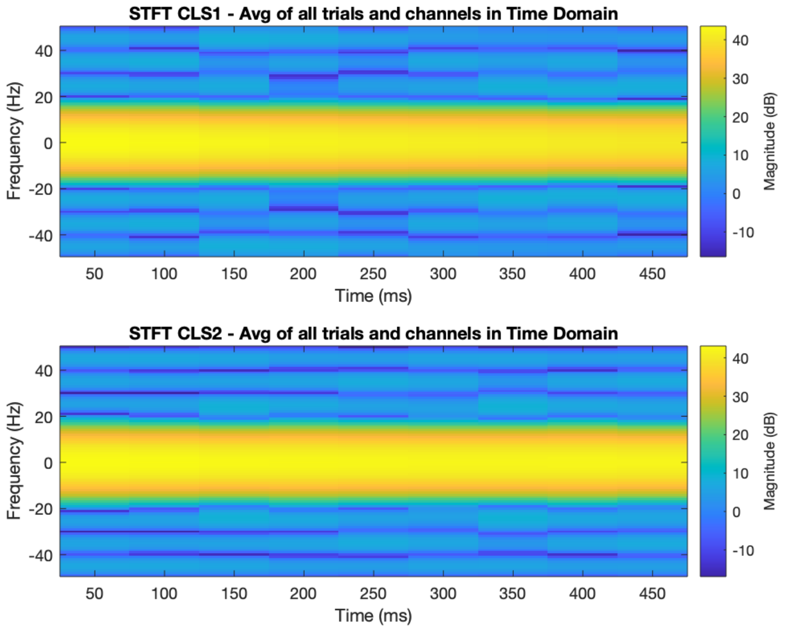


(b) STFT of Average of all Trials and all Channels

Figure 1: Amplitude and STFT of Average of all Trials and all Channels

I plotted these figures to first know more about the datas. Some times there is a very bold difference in FFT or amplitude of the signals which can be a good feature.

# 3 Features

I Extracted the following features:

## 3.1 Mean Frequency

## 3.2 Median Frequency

## 3.3 Total Power of Channels

## 3.4 Powers of Delta, Theta, Alpha, and Beta Frequency Bands

## 3.5 Entropy

## 3.6 Lyapunov Exponent

## 3.7 Average of Differentiate of Trials

## 3.8 Skewness

## 3.9 Kurtosis

## 3.10 Phase of the coefficients of the FFT

# 4 Fisher Score

Using fisher score, I selected good features for further steps. By calculating fisher score of all the features, I removed the features which their fisher score was less than $2 \times Avg(all\ of\ the\ fisher\ scores)$. Then using a greedy search, I selected 16 features which has the biggest ND fisher score. Using these 16 features, I performed classification using MLPs and RBFs.

# 5 Processing the Datas

I used CSP to maximize variance between the classes.

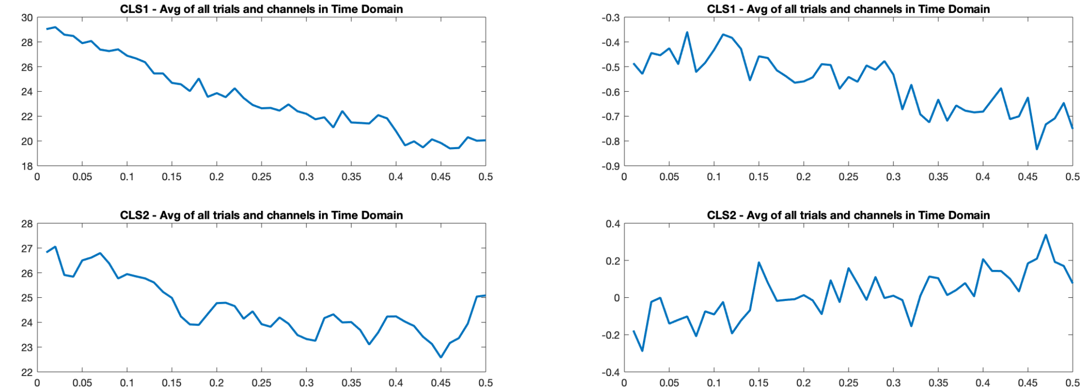


(a) Before CSP (b) After CSP

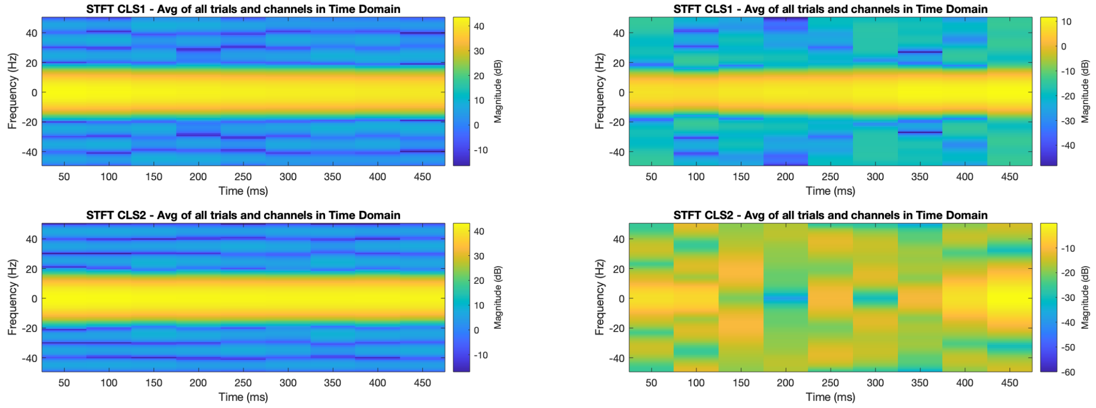


(c) Before CSP (d) After CSP

Figure 2: Amplitude and STFT of Average of all Trials and all Channels Before and After CSP

I stored the spatial filter $W$ for applying it on the test datas.
